# Normative reference equations of airway dynamics assessed by whole‐body plethysmography during spontaneous breathing evaluated in infants, children, and adults

**DOI:** 10.14814/phy2.15027

**Published:** 2021-09-13

**Authors:** Richard Kraemer, Hans‐Jürgen Smith, Heinrich Matthys

**Affiliations:** ^1^ Centre of Pulmonary Medicine Hirslanden Private Hospital Group Salem‐Hospital Bern Switzerland; ^2^ Department of Paediatrics University of Bern Bern Switzerland; ^3^ Department of Biomedical Research University of Bern Bern Switzerland; ^4^ Medical Development Research in Respiratory Diagnostics Berlin Germany; ^5^ Department of Pneumology University Hospital of Freiburg Freiburg Germany

**Keywords:** effective specific airway conductance, effective specific airway resistance, reference equations, specific aerodynamic work of breathing, whole‐body plethysmography

## Abstract

Effective specific airway resistance (sR_eff_), its reciprocal the effective specific airway conductance (sG_eff_) are computed as ratios between the integral of the resistive aerodynamic work of breathing (sWOB) and the integral of the tidal flow/volume loop, the reciprocal, respectively. Unfortunately, reference equations to obtain normative values for sR_eff_, sG_eff_, and sWOB are not yet available. To assess reference equations for sWOB, sR_eff_, and sG_eff_ during tidal breathing at resting level in healthy infants, children, and adults by a multidimensional model. Retrospectively exported data were collected from databases of five Swiss lung function centers, in which plethysmography (Jaeger Würzburg, Germany) was performed for the assessment of airway dynamics, static lung volumes, and forced breathing flow‐volume loops, in a collective of 28 healthy infants, 47 children, and 273 adults. From this cohort, reference equations were computed based on anthropometric measures, lung volumes, indices of the breathing pattern, and timing of breathing. By multi‐linear modeling reference equations of sR_eff_, sG_eff_, and sWOB could be defined taking as independent parameters apart from anthropometric parameters, also parameters given by the ratio between the tidal volume and functional residual capacity (FRC_pleth_/*V*_T_), and the ratio between *V*_T_ and inspiratory time (*V*_T_/*T*_I_). An alternative statistical approach to define reference equations of airway dynamics reveals that apart from the subject's anthropometric measurements, parameters of the magnitude of static lung volumes, the breathing pattern, and the timing of breathing are co‐variants of reference equations of airway dynamics over a large age range.

## Graphical abstract

By the work presented, the gap of the effectiveness regarding the embodiment of airway dynamics in the assessment of functional severity in various diseases can be bridged.

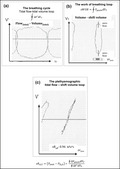

## 1 INTRODUCTION

Predictive equations defining reference values of lung function in humans are usually based on the subject's anthropometric measurements, such as age, body weight, body height or a combination of the above, as independent variables and gender. Most of these regressions include linear, but also power‐ or quadric function relationships (Hankinson et al., 1999). This may be suitable for some predictions of static lung volumes (Quanjer, Hall, et al., 2012; Quanjer, Stanojevic, et al., 2012), volume‐time or flow‐volume parameters (Hankinson et al., 1999; Quanjer et al., 1993), and indices of intrapulmonary gas distribution (Kraemer & Meister, 1985; Kraemer et al., 1986; Lum et al., 2013). A specific new approach to describe reference ranges more accurately was developed recently (Stanojevic et al., 2008), describing the relationship between spirometric lung function, height, and age, within the Paediatric age range, allowing a seamless transition to adulthood. An extension of the so‐called LMS (lambda, mu, sigma) method (Cole & Green, 1992; Cole et al., 2009) was applied. Prediction models using LMS‐statistics have been formulated for spirometric parameters (Quanjer, Stanojevic, et al., 2012), the carbon monoxide transfer factor (Stanojevic et al., 2017), and static lung volumes in individuals of European ancestry (Hall et al., 2021). However, there is a striking dearth of normative reference values availability measuring parameters of airway dynamics computed throughout the whole respiratory cycle during spontaneous breathing at rest such as the effective specific airway resistance (sR_eff_), its reciprocal value, the effective specific airway conductance (sG_eff_), and the specific aerodynamic work of breathing at rest (sWOB). The work of breathing (WOB) necessary to overcome the aerodynamic resistance of the airways during a breathing cycle (*R*_eff_) needs the integration of the mean ventilated lung volume generally approximated by FRC + *V*_T_/2 in the denominator of the formula for sWOB, sR_eff_, and sG_eff_.

**FIGURE 1:**
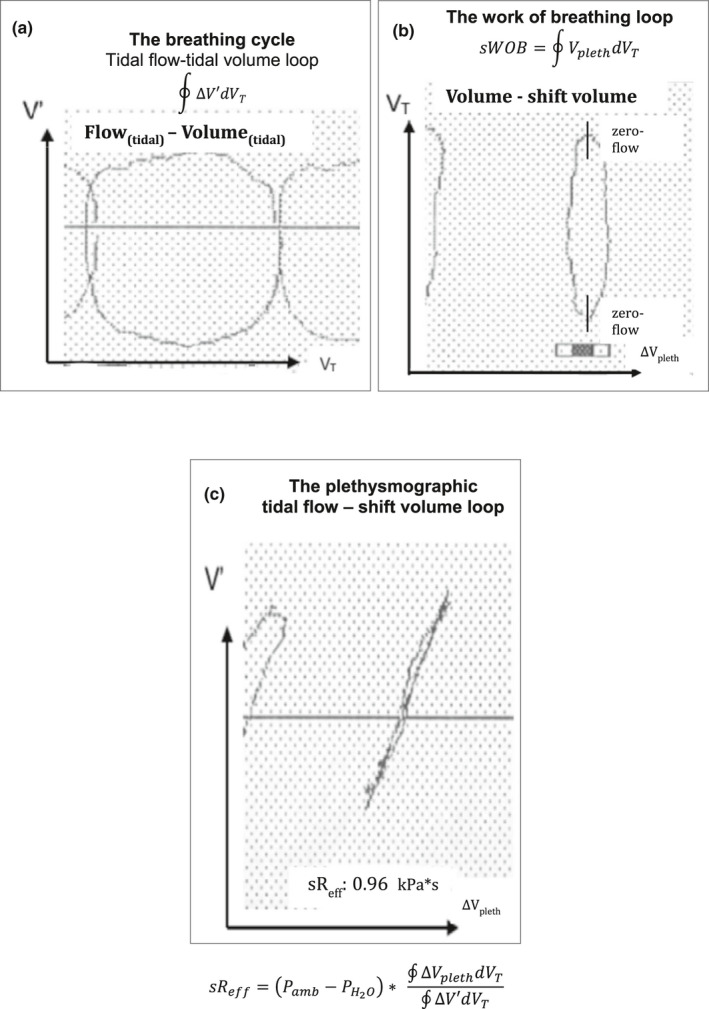
Print‐screen from a plethysmographic measurement of a healthy infant synoptically representing a BTPS‐compensated so‐called specific airway loop, (shift volume (∆*V*_pleth_)) against the tidal flow (*V*′) (c), derived from the tidal flow‐volume loop and its area as integral $\oint \Delta V^{\prime }dV_{T}$ (a), and the tidal volume in relation to the plethysmographic shift volume, incorporating the area $\oint \Delta V_{\text{pleth}}dV_{T}$, and hence the sWOB (b). sWOB is the fundamental prerequisite for the computation of sR_eff_, sG_eff_, respecively (c)

Although numerous parameters of airway resistances can be calculated from plethysmographic measurements (Ulmer et al., 1988), the most promising approach was proposed by Matthys and Orth (1975), defining the so‐called “effective specific resistance” (sR_eff_) as a ratio of the area of the plethysmographic shift‐volume versus tidal volume ($\oint V_{\text{pleth}}dV_{T}$), to the area of the flow/volume loop ($\oint V^{\prime }dV_{T}$) throughout the entire respiratory cycle. Noteworthy, the integral $\oint \Delta V_{\text{pleth}}dV_{T}$ multiplied by the dry air pressure ($P_{\text{amb}}‐P_{H_{2}O}$) embodies the specific, aerodynamic work of breathing (sWOB) at rest (Matthys & Orth, 1975). Only advanced computer technology made it possible to assess the two integrals presented for infants, children (Kraemer, 1993; Kusenbach et al., 1998), as well as for adults (Matthys & Orth, 1975; Zaiss & Matthys, 1990) according to the following equation:

$$sR_{\text{eff}}=P_{\text{amb}}‐P_{H_{2}O}*\frac{\oint \Delta V_{\text{pleth}}dV_{T}}{\oint V^{\prime }dV_{T}}=\frac{1}{sG_{\text{eff}}}$$

where ($P_{\text{amb}}‐P_{H_{2}O}$) is the dry air pressure, the integral $\oint V_{\text{pleth}}dV_{T}$ the equivalent to the area enclosed by sWOB and the integral $\oint V^{\prime }dV_{T}$ the equivalent to the area of the flow‐volume loop. Figure 1 synoptically represents a print‐screen from a plethysmographic measurement of a healthy infant obtained in the Jaeger infants whole‐body plethysmograph (Kraemer, 1993). It shows as first loops the tidal flow‐volume curves (a), and its area as integral $\oint \Delta V^{\prime }dV_{T}$ of each breathing cycle. The second loop (b) characterizes the tidal volume in relation to the plethysmographic shift volume and incorporates the area $\oint V_{\text{pleth}}dV_{T}$, and hence the sWOB. For the calculation of sWOB, the condiction that the zero‐flow points are in a close vertical relationship (BTPS corrected) must be fulfilled, for an appropriate sR‐loop (c), from which sR_eff_ and sG_eff_ can be computed according to the above‐mentioned equation. However, normative predictive equations of airway dynamics, such as sR_eff_, sG_eff_, and sWOB, are yet to be clearly defined.

A first attempt for measuring airway resistance throughout the whole respiratory cycle in infants was performed by Beardsmore et al. (1986), demonstrating a dynamic performance of the respiratory circuit in relation to the breathing pattern, and suggesting that the expiratory opening of the airway resistance loop (sR‐loop), what means that the expiratory part of the sR‐loop is opened due to different time constants of the emptying airflow, could well be due to small airway closure, as previously observed by Matthys (1973) in adults.

In order to predict gender‐specific normative values transitionally from infancy, through childhood into adulthood, we found it ground‐breaking to integrate into the regression analysis a part from anthropometric measurements, some important previously formulated findings of interrelationships in lung physiology (Beardsmore et al., 1986; Dominelli et al., 2015; Green et al., 1974; Hesser et al., 1990; Matthys, 1973; Mead, 1980; Sheel et al., 2009; Stocks et al., 2001). On one hand, we hypothesized that it is suitable to respect the disproportionate, but physiologically normal and gender‐specific growth between airways and lung parenchyma, a phenomenon defined as “dysanapsis” by Green et al. (1974), which may interfere with defining sWOB. It is well known that there are significant male–female differences in the luminal areas of the larger central and upper central airways (Jaeger & Matthys, 1968; Mead, 1980; Sheel et al., 2009), which are not accounted for by differences in lung size. On the other hand, our approach was accompanied by the findings of Hesser et al. (1990) investigating the interrelationship between end‐expiratory lung volume (EELV), tidal volume (*V*_T_), inspiratory (*T*_I_), and expiratory (*T*_E_) time during exercise in humans. These authors demonstrated a relationship between EELV and *T*_I_ with increasing work intensity (Lind & Hesser, 1984b). Our suggestion was, that this phenomenon could well be also an interaction to play a role during tidal breathing. Although the above‐mentioned observations were evaluated during exercise and different ambient conditions, we hypothesized that the pattern of breathing could significantly influence the measurements of airway dynamics at rest and be an important determinant for defining reference equations and hence normative values in humans during tidal breathing at rest. These findings of significant coherence in the interrelationship between lung size and breathing characteristics under which airway dynamics are measured, prompted us to evaluate reference equations using a multi‐level modeling.

Therefore, the purpose of the present investigation was to define gender‐specific normative reference equations of airway dynamics (sWOB, sG_eff_, sR_eff_,) by an alternative approach, incorporating (a) anthropometric measures (age, height, weight), potential influencing factors in relation to (b) the interrelationship to static lung volumes such as total lung capacity (TLC), vital capacity (VC), functional residual capacity (FRC_pleth_), and residual volume (RV), (c) the pattern of breathing such as the breathing frequency (BF), the tidal volume (*V*_T_), *V*_T_/FRC), and (d) the timing of breathing such as inspiratory time (*T*_I_), expiratory time (*T*_E_), (*V*_T_/*T*_I_, *V*_T_/*T*_E_), in healthy infants, children, and adults by multi‐regression models.

## 2 METHODS

### 2.1 Ethical approval

The study was planned according to the Federal Law of Human Research, conceptualized according to the Swiss Ethics Committees on research involving humans, and was conducted in accordance with the tenets of the Declaration of Helsinki. The study is part of the framework of the project entitled “Functional diversification of the Asthma‐ACO‐COPD multi‐center study” (ID 2017‐00259), approved by the Governmental Ethics Committees of the State of Bern, the State of St. Gallen, and the State of Zürich (Project KEK‐BE PB_2017‐00104). Written informed consent was waived because of the retrospective study design, which is following the institutional and national policies concerning research approvals. Master‐files haven been stored and secured in the REDCap‐system of the Clinical Trial Unit, Medical Faculty, University of Berne, Switzerland.

### 2.2 Participants

Plethysmographic data from healthy 38 infants (24 males, 14 females), 44 children (23 males, 21 females), and 270 adults (72 males, 198 females) were exported from five databases (University Children's Hospital): infants assessed by the Infant‐MasterLab children and adults assessed by the MasterLab (Division of Respiratory Medicine, Department of Paediatrics, University of Bern, Switzerland, Lung Centre of the Hirslanden Hospital Group, Berne; Clinic of Pneumology, Cantonal Hospital St. Gallen, Switzerland, Centre of Pulmonary Medicine, Hirslanden Private Hospital Group). The subjects from whom data were obtained were healthy infants and children participating in an epidemiologic study, healthy, no‐smoking lab technicians, students, volunteers, hospital staff, children of hospital staff, parents of children studied, and healthy participants of lung function instruction courses.

Inclusion criteria were reproducible baseline measurements with (a) at least five sR‐loops (shift volume‐tidal volume loops) of comparable shapes, (b) especially closed at zero flow points, with (c) closed expiratory part. Moreover, (d) inspiratory capacity (IC) has to be within the range of normal (Quanjer et al., 1993; Zapletal et al., 1987), in order to have achieved correct TLC and VC.

### 2.3 Pulmonary function procedures

Apart from an infant plethysmograph for the age group1 to 3 years (Master BabyLab, Jaeger) (Kraemer, 1993), in each center, the same procedure of lung function testing was performed, using a constant‐volume body plethysmograph (Master Screen Body, Jaeger) according to current recommendations (Stocks et al., 2001). Infants were sedated using chloral hydrate for sleeping within the infant plethysmograph (Kraemer, 1993; Rosenfeld et al., 2013), lung function testing was performed with the patient in a seated position. (Goldman et al., 2005). In the 1st phase, the assessment of airway dynamics (sWOB, sG_eff_, and sR_eff_) was obtained during at least 8 to 10 quiet breathing cycles (no panting). Since the integral method evaluates changes in airway dynamics concomitantly with changes in the EELV at FRC, it is important that parameters of airway dynamics are assessed in the first phase of plethysmographic assessment, and hence not influenced by deep inspiration or forced breathing maneuvers or other efforts (Kapsali et al., 2000; Salome et al., 2003; Slats et al., 2007). In the 2nd phase, the measurement of FRC_pleth_ at EELV was measured by at least three shutter closure maneuvers of comparable shapes, providing FRC‐volumes within a range of 7%. Except in infants, this 2nd phase was directly followed by a 3rd phase, the measurements of static lung volumes obtained by maximal expiratory and inspiratory breathing effort in order to calculate RV, VC, IC, and TLC. Thereafter, in a 4th phase at least three forced breathing maneuvers were performed measuring forced expired volume in 1 s (FEV_1_), forced vital capacity (FVC), maximal flows at 25% (FEF_25_), 50% (FEF_50_), and 75% (FEF_75_) of FVC, as well as the mid‐flow between 25% and 75% of forced expired lung volume (FEF_25–75_), assessed by standard techniques (Hankinson et al., 1999; Pellegrino et al., 2005; Stocks et al., 2001). For the plethysmographic measurements the median of at least five single plethysmographic shift volume—tidal flow loops were calculated, and for the indices of the forced breathing parameters the maximum of the three valid measurements was taken, as soon as the best and second‐best flow‐volume loops were comparable in their pattern. Pulmonary function test data of all parameters were assessed in absolute values, as the percentage of predicted normal values, and z‐transformed accordingly (Pellegrino et al., 2005; Stocks & Quanjer, 1995). The same standardized calibrations of flow, box leakage, and internal box pressure were performed daily in each center in the morning and at mid‐day, and a so‐called “biological calibration” was performed monthly using a healthy technician as a biological control. A special export software was developed by PanGas Ltd, Dagmersellen, Switzerland, enabling access to all routinely stored parameters in each JLab, Sentry‐Suite database resp.

### 2.4 Airway dynamics

Until recently, and still used in many plethysmographs, sR‐loops are approximated routinely by two point analyses creating a straight line throughout the loop.

$$sR_{\text{aw}}=\frac{\Delta V_{\text{pleth}}}{V^{\prime }}\cdot \left(P_{\text{amb}}‐P_{H_{2}O}\right)$$

Thereafter, functional residual capacity (FRC_pleth_) as corresponding absolute volume is measured by a shutter maneuvre and analyzing the occlusion‐pressure curve.

$${\text{FRC}}_{\text{pleth}}=\frac{\Delta V_{\text{pleth}}}{P_{m}}\cdot \left(P_{\text{amb}}‐P_{H_{2}O}\right)$$

Subsequently, airway resistance (Raw) can be calculated as a ratio of sRaw and FRC_pleth_.

$$R_{\text{aw}}=\frac{sR_{\text{aw}}}{{\text{FRC}}_{\text{pleth}}}=\frac{\Delta V_{\text{pleth}}\cdot P_{m}}{V^{\prime }\cdot \Delta V_{\text{pleth}}}\cdot \frac{\left(P_{\text{amb}}‐P_{H_{2}O}\right)}{\left(P_{\text{amb}}‐P_{H_{2}O}\right)}=\frac{P_{m}}{V^{\prime }}$$

It follows that *R*_aw_ is inevitably linked to the value of FRC_pleth_ as a fixed parameter in the equation, and is computed misleadingly low, if pulmonary hyperinflation is present, and consistently high, if the subject breathes under his natural EELV. Noteworthy is that FRC_pleth_ implicates a shutter occlusion maneuver, which is sometimes not tolerated by young children or elderly patients, as well as by patients with severe lung involvement. Moreover, *R*_aw_ consistently ignores the loop‐shaping of the sR‐loop, especially during quiet breathing, and more importantly, if ventilation inhomogeneities are present.

The advantage of this integral method compared with parameters of the two‐point analysis defining sR_aw_, sG_aw_, respectively, is, that data points throughout the entire respiratory cycle are evaluated. Moreover, the integral $\oint \Delta V_{\text{pleth}}dV_{T}$ embodies the specific, aerodynamic work of breathing (sWOB) at rest.(Matthys & Orth, 1975).

### 2.5 Mathematical and statistical approaches

To define the mathematical relationship between each lung function parameter as dependent parameters to be predicted, we first used the “curve estimation” tool of the Statistical Package for Social Science (SPSS, version 25, IBM) for linear, logarithmic, power and exponential regressions, as well as quadratic and cubic function for age, height, and weight, as previously proposed (Hankinson et al., 1999). It turned out, that most mathematical relationships featured power associations. Therefore, our modeling used absolute values and their natural logarithm (ln). The predicting equations for parameters of the breathing pattern (BF, *V*_T_), minute ventilation (MV), and the timing of breathing (*T*_i_, *T*_E_, *V*_T_/*T*_I_) presented gender differences with ln(age). For the evaluation of the reference equations of sWOB, sG_eff_, and sR_eff_ a multilevel linear model with a two‐level hierarchy was used, in detail given in the result section.

## 3 RESULTS

### 3.1 Measurement characteristics

The initial number of participant's measurements, exported from the five databases, suitable according to the inclusion criteria for the present study to be analyzed in a common merged database was 484. The screening regarding internal consistency and potential error values revealed that 0.8% of the measurements of static lung volumes had either FRC_pleth_, TLC, or IC beyond or above two standard deviations (SD), suggesting that these were measured incorrectly, mostly due to insufficient subject cooperation. In 4.7% the recoding of the FEV_1_, or FEF_25–75_ were out of the 2‐SD‐range. In another 0.5% of measurements the indices of timing the ratio between inspiratory time and expiratory time (*T*_I_/*T*_E_), as well as the ratio between inspiratory time and total time in one breath (*T*_I_/T_tot_) presented incomprehensible data, and finally in 2.3% of measurements sWOB data were measured or calculated erroneously, mostly due to an incoherent breathing technique. All these measurements were judged unsuitable for further evaluation. The study collective, therefore, consisted of 352 measurement sets (71.7% of all initially exported) including 38 infants (24 males, 14 females), 44 children (23 males, 21 females), and 270 adults (72 males, 198 females). There were more female subjects than males (females, mostly hospital staff members, were more easily recruited for such analysis than males). Apart from the infants and children, the age‐distributions in the centers were similar.

### 3.2 Pattern of breathing and timing

The breathing pattern and timing of breathing transitionally evaluated from infancy, over childhood into adulthood was evaluated for several parameters such as the BF, *V*_T_, MV, the inspiratory and expiratory time (*T*_I_, *T*_E_), and the ratio between *V*_T_ and *T*_I_ (*V*_T_/*T*_I_).

**FIGURE 2:**
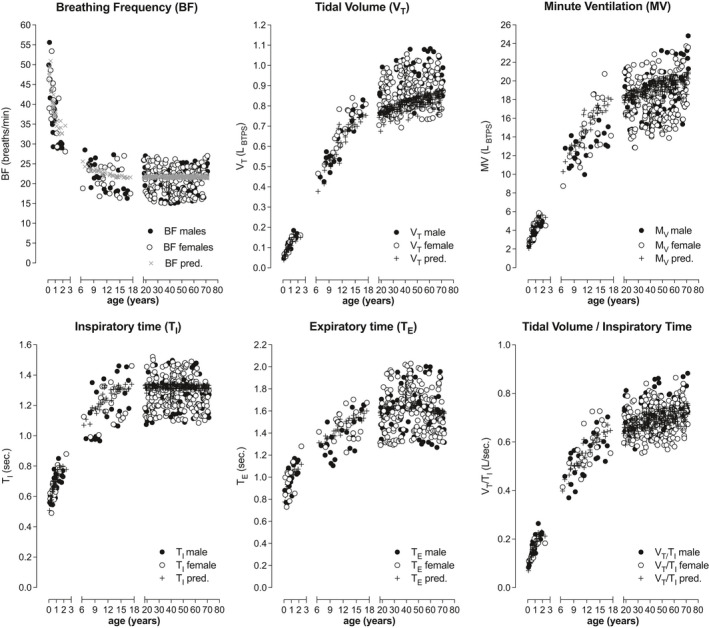
The breathing pattern and timing of breathing transitionally evaluated from infancy, over childhood into adulthood featuring normative predictive equations for the breathing frequency (BF), tidal volume at end‐expiratory level (*V*_T_), minute ventilation (MV), inspiratory time (*T*_I_), expiratory time (*T_E_*), and the ratio between *V*_T_ and *T*_I_ (*V*_T_/*T*_I_)

In Figure 2, the correlations and their regression equations between the breathing parameters and age from infancy, over childhood into adulthood are given and predicted values of these complex equations are plotted to visualize the predicted relationship. The splitting into three age function groups was performed in order to present the distributions best optically. The regression equations, however, are computed over the whole age range from birth to 80 years and are given in Table 1. The BF decreased from 37.9 ± 7.2 breaths per minute in infancy to 20.3 ± 3.2 breaths per minute during childhood remaining at 20.2 ± 3.2 breaths per minute during adulthood. Conversely, *V*_T_ increased gradually from 0.108 ± 0.037 L in infancy to 0.65 ± 0.121 L during childhood to 0.900 ± 0.084 L in adulthood. Similar changes were also found for the timing *T*_I_ which increased from 0.672±0.088 s during infancy, to 1.271 ± 0.106 s during childhood, and remained at 1.295 ± 0.119 s at adulthood. Similarly, also *T*_E_ increased from 0.979 ± 0.126 s to 1.420 ± 0.159 s during childhood, 1.656 ± 0.205 s, during adulthood resp. Combining the breathing pattern with the timing of breathing showed, that *V*_T_/*T*_I_ increased from 0.157 L/sec to 0.553 ± 0.091 L/sec during childhood to 0.693 ± 0.063 L/sec during adulthood. Surprisingly, some values of especially *V*_T_ are rather high, presumably to the circumstance, that the subjects are enclosed in a body box, a condiction which could stimulate the subjects to breath deeper. This emotional aspect is more pronounced in adults than children.

**TABLE 1:** Normative regression equations of the breathing parameters: breathing frequency (BF), resting tidal volume (*V*_T_), minute ventilation (MV), and the parameters of the timing of breathing inspiratory and expiratory time (*T*_I_, *T_E_* resp.), as well as the ratio between *V*_T_ and *T*_I_ (*V*_T_/*T*_I_) evaluated as transitional computation from infancy over childhood to adulthood

BF = EXP ((10.898) + (0.009*Gender) + (0.010*Height) + 1.878*Ln(Height))
Gender: 0 = males (*n* = 119), 1 = females (*n* = 233); Height in cm
*V*_T_ = EXP ((−5.388) + (−0.007*Gender) + (0.101*Ln(Age) + (0.054*Height)) + (−0.000151*Height^2^))
Gender: 0 = males (*n* = 119), 1 = females (*n* = 233); Age in years; Height in cm
MV = EXP ((−0.534) + (0.010*Gender) + (0.098*Ln(Age)) + (0.033*Height) + (−0.000087*Height^2^))
Gender: 0 = males (*n* = 119), 1 = females (*n* = 233); Age in years; Height in cm
*T*_I_ = EXP ((−1.595) + (0.020*Gender) + (−0.021*Height) + (−0.000059*Height^2^))
Gender: 0 = males (*n* = 119), 1 = females (*n* = 233); Height in cm
*T*_E_ = EXP ((−0.160) + (0.029*Gender) + (0.092*Ln(Age)) + (−0.000026*(Age^2^) + (0.002*Height)))
Gender: 0 = males (*n* = 119), 1 = females (*n* = 233); Age in years; Height in cm
*V*_T_/*T*_I_ = EXP ((−10.431) + (−0.015*Gender) + (0.085*Ln(Age)) + (2.040*Ln(Height)) + (−0.000025*Height^2^))
Gender: 0 = males (*n* = 119), 1 = females (*n* = 233); Age in years; Height in cm

### 3.3 Influence of volumetric and timing indices of ventilation to airway dynamics

**FIGURE 3:**
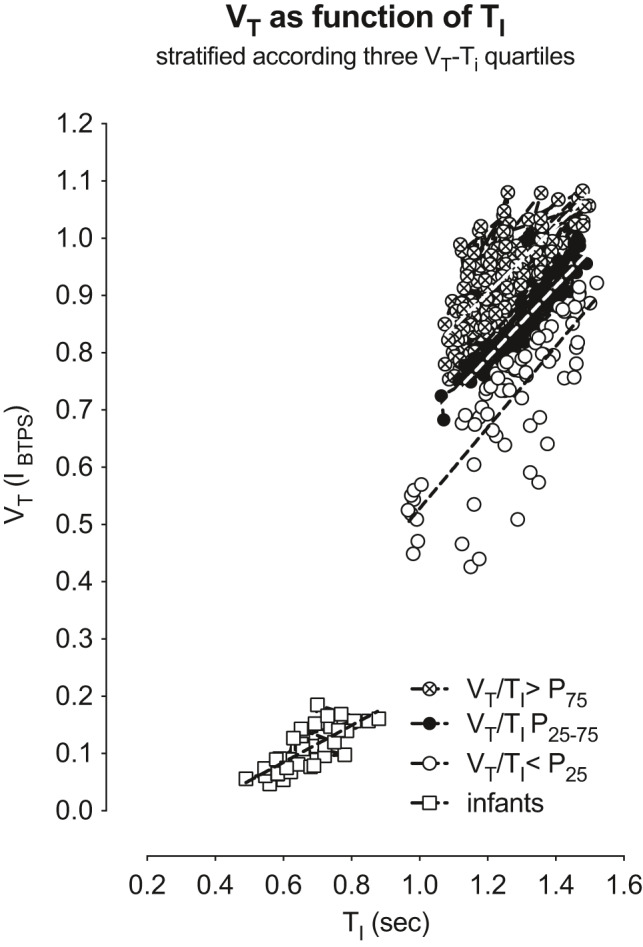
Tidal volume (*V*_T_) as a function of inspiratory time (*T*_I_) within quartiles of *V*_T_/*T*_I_ at rest

In analogy to Hesser's figure 5 (Hesser et al., 1990) showing good correlations between *V*_T_ and isopleths for different *V*_T_/*T*_I_ during exercise, we can demonstrate, that similarly significant regressions between *V*_T_ and *T*_I_ are related within quartiles of *V*_T_/*T*_I_ during resting tidal breathing. Figure 3 shows that as *T*_I_ increased from infancy to adulthood, *V*_T_ increased within the three *V*_T_/*T*_I_ quartiles. The linear regressions between *T*_I_ and *V*_T_/*T*_I_ were significantly different (*p* < 0.001) with slopes of 0.322 ± 0.047, 0.708 ± 0.058, and 0.584 ± 0.044 within the *V*_T_/*T*_I_ quartiles *p*_<25_, *p*_25–75_, and *p*_>75_, respectively. Quartiles were chosen in order to quantify the ratio of *V*_T_/*T*_I_ to which no values predicted are available. Based on these and previous observations that the threshold of the inspiratory off‐switch mechanisms depends on central inspiratory activity (Von Euler, 1983), which in turn increases with airway resistance (Hesser & Lind, 1984), we hypothesized that the volumetric and timing indices of ventilation could well influence the measurements of airway dynamics even at rest. Therefore, *V*_T_/*T*_I_ must be included as a parameter when defining normative values und predictive equations in humans over all age ranges. This remarkable coherence of interrelationships between breathing characteristics and timing of breathing prompted us to study the parameters of lung dynamics sWOB, sG_eff_, and sR_eff_ at rest, in relation to different parameters defining respiratory output, the breathing pattern, and the EELV, under which conditions these parameters are measured (Stocks et al., 2001).

### 3.4 Predictive data analysis of airway dynamic parameters

In order to search for true interactions and for confounding parameters defining mainly the specific aerodynamic work of breathing, a multilevel linear model with a two‐level hierarchy was used. In level 1, potential influencing parameters of “*anthropometry*,” “*lung volumes*,” “*the breathing pattern*,” and “*the timing of breathing*” was evaluated resolving multicollinearity by stepwise regression of ANOVA within these four “*function groups*,” as follows:

- the *anthropometric function group* containing age, ln(age), age^2^, height, ln(height), height^2^, weight, ln(weight) and weight^2^,
- the *function group of static lung volumes* containing TLC, ln(TLC), VC, ln(VC), IC, ln(IC), FRC_pleth_, ln(FRC_pleth_), RV, ln(RV), FRC_pleth_/TLC, IC/TLC, RV/TLC, and *V*_T_/FRC_pleth_,
- the *function group of the breathing pattern* given by BF, ln(BF), *V*_T_, ln(*V*_T_), MV, ln(MV), and
- the *function group defining respiratory timing T*_I_, ln(*T*_I_), *T*_E_, ln(*T*_E_),*T*_I_/*T*_tot_, *T*_E_/*T*_tot_, *V*_T_/*T*_I_ ln(*V*_T/_ *T*_I_), *V*_T_/*T*_E_, ln(*V*_T/_ *T*_E_).

Multicollinearity statistics were checked by the *t*‐*statistics*, and the *variance inflation factor (VIF)* as a predictor of the linear relation to the dependent variable, (VIF < 5). The stepwise variable selection has, similarly to the forward variable selection, the advantage that after a variable is entered, all variables already in the model are re‐examined to see if any of them meet the criteria for removal. Apart from the actual *F* change the entry criterion was a *p* ≤ 0.05, for removal a *p* ≤ 0.10. Applying least squares (OLS) statistics, a minimum‐variance mean‐unbiased estimation and hence the prediction of first sWOB, and sG_eff_, and sR_eff_ could be obtained.

Since we are faced with the potential problem of multicollinearity, the selection of parameters suitable for the final overall regressions was made based on “tolerance of collinearity” defined as 1 minus *R^2^* and the “variance inflation factor” (VIF) defined as reciprocal of the tolerance. Parameters with values of tolerance less than 0.2 and values of VIF higher than 10 were considered problematic and stepwise excluded.

The procedure of stepwise exclusion of parameters within the four function groups was performed as follows:

**Table:** 

1st function group:	**sWOB in relation to parameters of “*anthropometry*”** sWOB = EXP(−16.657 + 0.018*Gender + 0.257*ln(age) + 3.288*ln(height) − 3.807E‐5*height^2^) ± 0.143071 (SEE); male = 0; female = 1 excluded: age, age^2^, height, height^2^ (*F*‐value: 3837, *p* < 0.0001; accuracy: 97.3%).
2nd function group:	**s** **WOB in relation to “** ***static lung volumes and*** *V*_T_ ***at EELV*** **”** sWOB = EXP(−0.452 + 0.009*Gender + 0.144*ln(age) + 1.096*ln(FRC_pleth_) + 0.980*ln(*V*_T_/FRC_pleth_)) ± 0.113399 (SEE); male = 0; female = 1 excluded: FRC_pleth_, FRC_pleth_/TLC (*F*‐value: 6160, *p* < 0.0001; accuracy: 97.2%).
3rd function group:	**s** **WOB in relation to parameters of “** ***breathing pattern”*** sWOB = EXP(−1.124 + 0.001*Gender + 0.133*ln(age) + 1.221*ln(*V*_T_) + 0.293*ln(BF)) ± 0.106902 (SEE); male = 0; female = 1 excluded: BF, *V*_T_, MV, ln(MV) (*F*‐value: 6943, *p* < 0.0001; accuracy: 96.2%).
4th function group:	**s** **WOB in relation to parameters of “** ***timing of breathing*** **”** sWOB = EXP(−0.222 + 0.012*Gender + 0.151*ln(Age) + 1.218*ln(*V*_T_/*T*_I_) + 0.817*ln(*T*_I_)) ± 0.109582 (SEE); male = 0; female = 1 excluded: *T*_E_, ln(*T* *_E_*), *V*_T_/*T* *_E_*, ln(*V*_T_/*T* *_E_*), *V*_T_/*T*_I_, *T*_I_ (*F*‐value: 6603, *p* < 0.0001; accuracy: 96.2%).

Within each function group, diagnostic accuracy was verified by the automatic linear modeling (ALM) approach in SPSS (Yang, 2013).

In a second step, sWOB was predicted by summarizing all selected, potentially dependent parameters and combining the four function groups of the model. Assessing the “best” model for the data using marginal *t*‐tests for random effects and hence avoiding collinearity, we utilized the marginal test by primary forward, and checking by backward selection. Once we identified the parameters with the smallest marginal *p* values (<0.05) and good tolerance as well as VIF, we incorporated the identified parameters, selected from each model to a final equation, which was again regressed to the most significant parameters. From this combined model the regression equations (±SEE) for sWOB (kPa*L*L) then were as follows:

**Table:** 

**sWOB** = EXP(−0.300 + 0.017*gender + 0.138*ln(age) + 0.836*ln(FRC_pleth_) + 0.744*ln(*V*_T_/FRC_pleth_) + 0.387*ln(*V*_T_/*T*_I_)) ± 0.109083 (SEE) *gender: male = 0; female = 1(*F*‐value: 5332, *p* < 0.0001; accuracy: 97.3%)

Accordingly, sG_eff_ and sR_eff_ can be defined, using the same multi‐level models including sWOB as follows:

**Table:** 

**sG_ef_**_f_ = EXP(0.816 − 0.050*gender − 0.423*ln(sWOB) + 0.415*ln(FRC_pleth_) + 0.603*ln(*V*_T_/FRC_pleth_)) ± 0.12781 (SEE) *gender: male = 0; female = 1(*F*‐value: 134.1, *p* < 0.0001; accuracy: 53.8%)
**sR_ef_**_f_ = EXP(−0.816 + 0.050*gender + 0.423*ln(sWOB)−0.415*ln(FRC_pleth_) − 0.603*ln(*V*_T_/FRC_pleth_)) ± 0.12781 (SEE) *gender: male = 0; female = 1(*F*‐value: 134.1, *p* < 0.0001; accuracy: 53.8%).

## 4 DISCUSSION

### 4.1 Findings of the present study

The present study aimed to define normative regression equations of the relationship between parameters of airway dynamics, transitionally assessed by the plethysmographic integral method, obtained in healthy infants, children, and adults. The mathematical approach is based on the concept that sR_eff_, sG_eff_ resp. are derivatives of sWOB. To our knowledge, the very complex interaction of airway patency and the individual lung structure (dysanapsis) on one hand, and the individual breathing characteristics, on the other hand, was never studied before. International standards defining normative regression equations are only by few references available so far (Crieé et al., 2011; Goldman et al., 2005; Stocks et al., 2001). The major mathematical component of the equations defining sG_eff_ and/or sR_eff_ is sWOB, given by the integral of the plethysmographic shift volume versus its tidal volume loop ($\oint \Delta V_{\text{pleth}}dV_{T}$). This plethysmographic parameter, expressing the specific aerodynamic work of breathing at rest, has not yet reached enough attention so far. In addition to the gender‐specific power function to age, sWOB also depends on FRC_pleth_, the breathing pattern (*V*_T_/FRC), and the respiratory timing (*V*_T_/*T*_I_). This kind of modeling has shown to be predictive for a large age range beginning in infancy, through childhood into adulthood. The interrelationship between and the EELV and sWOB (and hence the parameters of airway dynamics sR_eff_ and sG_eff_) and the end‐expiratory level at FRC_pleth_ with its specificity what the breathing characteristics patter and the timing of breathing is concerned highlights the multifaceted variability of airway dynamics in healthy subjects. In fact, during normal breathing, the healthy human body disposes over a wide range of possibilities and may serve as a model of how the interactions would be in patients with respiratory disorders.

Therefore, the outcome of the present study reveals some advantages to assess airway dynamics in patients (Matthys, 1973; Matthys et al., 1970). In contrast to the older parameters sRaw and its derivatives, the estimation of the degree of airway patency assessed by sR_eff_ is closely measured in relation to the actual EELV, defining FRC. The older “straight line” definitions of sRaw and its derivatives are largely dependent on the actual chosen value for ∆V_pleth_ at a single given flow in the shift volume flow diagram (see figures and equation in the section airway dynamics). The disposal of parameters expressing airway dynamics throughout the in‐ and expiratory breathing cycle in relation to the actual EELV at FRC is particularly important, if the EELV is altered especially in patients with lung disease. This is essential for the assessment of changes in airway dynamics in relation to the distending forces of the thoraco‐pulmonary system, especially in patients with pulmonary hyperinflation (with or without trapped air) (Kraemer et al., 2006; Matthys et al., 1970), small airway disease, pneumothorax and/or extrathoracic airway obstruction such as vocal cord dysfunction, tracheal tumors, membrane restrictions. The interpretation of the shift volume—tidal flow loops can be more diagnostic than the numerical values of in‐ and expiratory, or total airway resistance parameters. Apart from this advantage, and in contrast to parameters requiring the instruction of forced breathing maneuvers, the plethysmographic measurements during spontaneous tidal breathing, are demanding little cooperation and are nearly effort independent. Moreover, the effect of deep inspiration and its influence on the regional distribution of the air and hence their impact on changes in the so‐called “volume history” (Agostoni & Mead, 1964; Duggan et al., 1990; Mead et al., 1967) can be avoided.

### 4.2 Paradigm‐change expressing airway dynamics

A major step in the assessment of airway dynamics throughout the entire plethysmographic shift volume—tidal flow loop (Figure 1) and its mathematical comprehension of loop shaping, was initially elaborated and introduced by Matthys and Orth (1975). The aim of this initial approach shortly coming up after the report of Islam and Ulmer (Islam & Ulmer, 1974), was to analyze the contribution of these pathophysiological disturbances to a dissociation between greatest shift volume to its corresponding flow. These authors extended the dimensional analysis applied by Jaeger and Otis (1964) to integrate these contributions to an **"**effective resistance**"** that included the effects of the entire range of variable flows during tidal breathing and nonlinearities in the breathing loop. The outstanding characteristic of sG_eff_, sR_eff_, respectively, is its reflection of an integrative assessment of airway behavior throughout the entire tidal breathing cycles. Moreover, the digital integration of the respective loops improves the signal‐to‐noise ratio.

The specific work of breathing (sWOB) can be considered as an approximation of the total gas‐dynamic (impedance) work performed during a breathing cycle (Matthys & Overrath, 1971). The energy requirement for normal resting breathing takes only a small fraction of the basal metabolism in healthy subjects, but may be of considerable magnitude in patients with obstructive pulmonary diseases (Otis, 1954). There are two components of the work of breathing during respiration: the flow‐aerodynamic work of breathing and the elastic work of breathing. The former refers to the work to overcome the frictional resistance to gas flow due to compression and decompression within the ventilated airways and the elastic components for tissue movement, whereas the latter overcomes the elastic recoil during inhalation storing energy to be recovered during expiration. Both, flow‐resistive and elastic work are conducted during inspiration and expiration. Classically, it may be computed in terms of esophageal pressure multiplied by the change in pulmonary volume.

Although the esophageal pressure measurement remains the solid reference technique to completely quantitate the efforts of breathing to move the lung, whole‐body plethysmography allows the estimation of the gas‐dynamic, resistive effort by referring the integral of the plethysmographic shift‐volume, and hence the intra‐plethysmographic pressure changes, to the integral of the tidal volume. In a constant volume whole‐body plethysmograph, the shift volume refers to the magnitude of lung volume which fades away in compression and originates in decompression and which is proportional to the airway resistance and the absolute, ventilated, and non‐ventilated lung volumes. Therefore, the specific gas‐dynamic work performed during resting tidal breathing can be estimated by the simultaneous assessment of the plethysmographic shift‐volume and the corresponding tidal volume (Figure 1).

### 4.3 Complexity‐defining prediction equations for airway dynamics

Airway resistance in humans increases as a power function of flow, and in close proportion to the square root of density (Maio & Farhi, 1967; Pedley et al., 1970). Previous work has shown that airway resistance is highly dependent upon the breathing pattern and the EELV, at least during exercise (Hesser et al., 1990). It was well demonstrated in the 1980s and 1990s that there is a significant relationship between the pattern of breathing and airway resistance during exercise, and that the threshold of the so‐called inspiratory off‐switch mechanisms must be taken into account with the central inspiratory activity (Hesser & Holmgren, 1959; Hesser et al., 1990). The pattern of breathing and airway resistance during exercise in terms of the relationships of inspiratory time (*T*_I_), the tidal volume (*V*_T_), and to EELV was extensively studied by Hesser and Lind (Hesser et al., 1990; Lind & Hesser, 1984a, 1984b), showing the interrelationship between *T*_I_, and *V*_T_ in different ranges. Our analyses (Figure 3) demonstrate that such interrelationships also play a role during tidal breathing, representing a physiologic argument for the inclusion of such parameters in the predicting equations.

### 4.4 Clinical relevance of this paradigm‐change

The multi‐level factor approach defining the reference values of airway dynamics in healthy subjects transitionally, could lead to a certain paradigm change, especially what dynamic tests in patients are concerned. The assessment of bronchodilator response (BDR) on one hand, and the assessment of airway hyperreactivity (AHR) by methacholine challenge test (MCT) on the other hand (Kraemer et al., 2016), are important diagnostic tools to differentiate various diagnostic groups such as asthma, chronic obstructive pulmonary disease (COPD), asthma‐COPD‐overlap (ACOS). Both test‐procedures – BDR and MCT – are principally based on defining changes of airway dynamics during these test procedures. However, these diagnostic procedures are mostly tested by forced breathing spirometric parameters. In parallel to the present report, a publication will be released presenting the fundamental differences between BDR assessed by spirometric parameters and BDR based on z‐score‐changes, obtained by plethysmographic parameters of airway dynamics in patients with chronic obstructive lung disease (Kraemer et al., 2021). Therefore, it could well be that regarding BDR, and thus reversibility of airway obstruction, or MCT, and accordingly the diagnostic tool for AHR, the specific aerodynamic parameters (sR_eff_, sG_eff_ and sWOB) could serve as better reliable parameters to define specific disease endo‐phenotypes.

## 5 CONCLUSION

There are many advantages using the plethysmographic parameters (sWOB, sG_eff_, sR_eff_,) obtained by the integral method over the whole range of the breathing cycle as objective target parameters of airway dynamics. Transitorily applied over the whole age range from infancy to adulthood by the present work, the gap in accurate regression equations to obtain individual normative data can be filled. Prospectively designed future studies are expected to demonstrate the potential worth for discernment offered by the normative equations of these target parameters.

## AUTHOR CONTRIBUTIONS

RK designed, coordinated, and analyzed the data, and drafted the manuscript. H‐JS gave advice on the technical parts of the data acquisition and took part in the interpretation of data and revising, and HM edited and revised the manuscript. All authors approved the final version of the manuscript.

## CONFLICT OF STATEMENT

The authors have no conflicts of interest to declare. The study was planned according to the Federal Law of Human Research, conceptualized by the Swiss Ethics Committee on Research involving humans, giving evidence that the research was conducted ethically in accordance with the World Medical Association Declaration of Helsinki. It was approved by the Governmental Ethics Committees of the State of Berne, Zürich, and St. Gallen. Master‐files haven been stored and secured in the REDCap‐system of the Clinical Trial Unit, Medical Faculty, University of Berne, Switzerland.
